# Efficient myoblast expansion for regenerative medicine use

**DOI:** 10.3892/ijmm.2014.1763

**Published:** 2014-04-30

**Authors:** DANUTA JAROCHA, KLAUDIA STANGEL-WOJCIKIEWICZ, ANTONI BASTA, MARCIN MAJKA

**Affiliations:** 1 Department of Transplantation, Polish-American Institute of Pediatrics, Jagiellonian University School of Medicine, 30-663 Cracow, Poland; 2 Department of Gynecology and Oncology, Jagiellonian University School of Medicine, 31-501 Cracow, Poland

**Keywords:** myoblasts, expansion, differentiation, cell replacement therapy

## Abstract

Cellular therapy using expanded autologous myoblasts is a treatment modality for a variety of diseases. In the present study, we compared the commercial skeletal muscle cell growth medium-2 (SKGM-2) with a medium designed by our group for the expansion of skeletal myoblasts. The use of an in-house medium [DMEM/F12 medium supplemented with EGF, bFGF, HGF, insulin and dexamethasone (DFEFH)] resulted in a greater number of myoblast colonies (>50%) and a 3-, 4- and 9-fold higher proliferation rate, eventually resulting in a 3-, 7- and 87-fold greater number of cells at the 1st, 2nd and 3rd passage, respectively, compared with the cells grown in SKGM-2 medium. The average CD56 expression level was higher in the myoblasts cultured in DFEFH than in those culturd in SKGM-2 medium. At the 3rd passage, lower expression levels of myostatin and considerably higher expression levels of myogenin were observed in the cells that were grown in DFEFH medium. The results of our study indicated that myoblasts cultured in both medium types displayed fusogenic potential at the 3rd passage. Furthermore, it was shown that cells cultured in DFEFH medium created myotubes with a considerably higher number of nuclei. Additionally, we observed that the fusion potential of the cells markedly decreased with the subsequent passages and that the morphology of the myoblasts differed between the 2 cultured media. Our data demonstrate that culture in the DFEFH medium leads to an approximately 90-fold greater number of myoblasts, with improved morphology and greater fusion potential, compared with culture in the commercial SKGM-2 medium.

## Introduction

Autologous myoblasts that have expanded from satellite cells isolated from muscle biopsy samples constitute the basis for cell replacement therapies in regenerative medicine for the treatment of urinary incontinence, ischemia-damaged myocardium or Duchenne muscular dystrophy. Clinical reports on autologous muscle-derived cells (MDCs) used for the treatment of either ischemia-damaged myocardium or urinary incontinence in humans have demonstrated the dependency of the therapeutic effect on the number of injected cells, with >1×10^8^ myoblasts required to obtain significant results or improved symptoms (1–3). These observations indicate that this treatment strategy may require a high number of competent cells to obtain a sufficient therapeutic effect.

Satellite cells remain in a quiescent state in uninjured tissue. Minor damage or injury to the tissue stimulates the release of hepatocyte growth factor (HGF) and basic fibroblast growth factor (bFGF) from injured myofibers (4,5). HGF activates satellite cells and causes them to enter the cell cycle, proliferate, differentiate and fuse to regenerate injured muscle fibers (4). HGF has been shown to be the only growth factor capable of activating satellite cells in *in vitro* primary culture (6–8). bFGF has been shown to enhance myoblast proliferation by increasing cyclin-D1 mRNA expression between 4 and 8 h post-induction with a return to initial levels by 32 h post-induction (9). Notably, bFGF has been reported to enhance the HGF-stimulated proliferation of myoblasts (10) and to repress the terminal differentiation of myoblasts (11). McGeachie and Grounds have shown *in vivo* the presence of dividing myoblasts up to 120 h after damage (12). However, this rate of proliferation is not maximal and can be increased *in vitro* by the addition of members of the fibroblast growth factor family (13,14). Epidermal growth factor (EGF), platelet-derived growth factor (PDGF) and tumor growth factor (TGF)-β have also been reported to enhance myoblast proliferation *in vitro* (15–17). When proliferating myoblasts must withdraw from the cell cycle to differentiate, growth factors, such as HGF and bFGF, which stimulate cell cycle progression, regulate the activity of myogenic regulatory transcription factors, such as MyoD, myogenic factor 5 (Myf5), myogenin and myogenic regulatory factor (MRF)4, that have been shown to control the specification and differentiation of the muscle lineage (18). During regeneration, activated satellite cells reportedly initially express either Myf5, MyoD or both (19,20). Myogenin is required for the differentiation of myoblasts (21); MRF4 is thought to be involved in the maturation of myotubes (22).

Myostatin, a growth factor and a TGF-β superfamily member, is a specific negative regulator of skeletal muscle mass (23). This growth factor has been shown to play a role in regulating the activation, growth and self-renewal of satellite cells (24) and to inhibit the growth of myoblasts (25). Myostatin has also been shown to negatively regulate myogenic differentiation by inhibiting the mRNA and protein expression of MyoD, Myf5, myogenin and myosin heavy chain 2A (MyHC-2A) (26,27). MyHC-2A is one of 3 fast-type isoforms of a muscle contractile protein known as myosin heavy chain (28). In low seeding density cultures without supplemental growth factors, MyHC-2A mRNA expression has been shown to increase in parallel with a decrease in Myf5 and myogenin expression; this result indicates a correlation with phenotypic differentiation (29).

Initial *in vitro* experiments with muscle cell progenitor cultures have been performed in Ham’s F10 or Ham’s F-12 media (30,31) and have also been performed in other media, such as Dulbecco’s modified Eagle’s medium (DMEM) (32,33). However, the use of these media results in a low number of cells. Published culture strategies aimed at increasing the number of obtained myoblasts have emphasized the importance of proteins used for flask covering, supplementation with various growth factors and different cell passaging strategies, as well as the effect of these variables on the kinetics and the proliferation potential of myoblast expansion (17,29,31,34–36). The effectiveness of EGF, FGF and PDGF growth factors in enhancing expansion capacity has also been reported (16,36). Thus, a higher number of myoblasts can be obtained using skeletal muscle cell growth medium (SKGM) (3,37) or DMEM with the addition of growth factors. A high proportion of serum and non-confluent culture conditions have been shown to prevent myogenic differentiation (38). An automated culture system indicated that the optimal seeding density and attainable confluence was 1×10^3^ cells/cm^2^ and 50% confluence, respectively (39). However, a recent study indicated that prolonged culture leads to an increased percentage of senescent cells, a decreased ability of myoblasts to form myotubes and alterations in glucose and lipid metabolism (40).

In the present study, we compared the commercial medium, SkGM™-2 BulletKit™Medium (SKGM-2; current version of the no longer available SKGM), with a medium designed by our group [DMEM/F12 medium supplemented with EGF, bFGF, HGF, insulin and dexamethasone (DFEFH)]. The aim of this study was to seek the optimal large-scale expansion conditions of functional MDCs for regenerative medicinal use by comparing the expansion efficacy and the fusogenic potential of skeletal myoblasts in these 2 media.

We demonstrate culturing conditions that produce up to 5×10^9^ myoblasts, presenting fusogenic potential in 24 days. We also demonstrate that the number of myoblast colonies, the doubling time, the duration of the logarithmic growth phase, the number of expanded cells and the cell morphology strongly depend on culture conditions. Our results demonstrated that the use of an in-house medium resulted in an approximately 100-fold greater number of cells of less differentiated morphology and higher fusion potential during 3 passages compared with cells cultured in SKGM-2 medium. Additionally, we show that the expansion of myoblasts over 23 generations results in a rapid decrease in the proliferation potential, and an increase in the doubling time, the appearance of vacuoles and the loss of the fusion potential of these cells.

## Materials and methods

### Muscle biopsy and primary cell culture

Deltoid muscle samples of approximately 0.2 g were acquired from women with stress urinary incontinence (20% available for the experiments), aged over 50 years, by a needle biopsy performed under local anesthesia. Both institutional review board approval and informed consent from all participants were obtained prior to the biopsy procedure. All muscle biopsies were transported in phosphate-buffered saline (PBS) with antibiotics (PAA Laboratories GmbH, Goetzis, Austria) and stored at room temperature until processing, which was generally performed 2 h after the biopsy had been obtained. First, the muscle samples were rinsed in PBS and cleaned by removing adherent fat and tendon tissues using a scalpel. The muscle tissue was then cut into small sections and digested with 2 mg/ml of collagenase 1A (Sigma-Aldrich, Seelze, Germany) for 40 min at 37°C, as previously described (33). Individual fibers were liberated by rigorous pipetting and were centrifuged at 300 × g for 5 min. The pellet was resuspended in a small volume of PBS, was divided into 2 parts and was centrifuged again.

To compare the expansion efficacy of the SKGM-2 medium (Lonza, Walkersville, MD, USA) (formulated by combining SKBM™-2 Basal Medium, CC-3246 with the SkGM™-2 SingleQuots™ kit, CC-3244) with that of the in-house medium, one-tenth of the muscle fibers was resuspended in SKGM-2 medium and another one-tenth was resuspended in the medium designed by our group: DMEM/F-12 (PAA Laboratories GmbH) supplemented with dexamethasone, insulin (both from Sigma-Aldrich), 18% fetal bovine serum (FBS) (PAA Laboratories GmbH) and the growth factors, EGF, FGF (2) and HGF, (DFEFH). The muscle fibers were then transferred into 6-well plates coated with laminin (Sigma-Aldrich) to enable the released satellite cells to adhere to or migrate out of the muscle fibers. The plates were incubated for 48 h in a humidified atmosphere containing 5% CO_2_. After 48 h, the medium with the muscle fragments that had not adhered to the plates was changed (P0) and the supernatant containing the muscle fibers was transferred into new laminin-coated wells (P1). After a further 48 h, the procedure was repeated and the medium in all wells was changed every 3 days. At day 8 of culture, the number of colonies, defined as a cluster of at least 10 cells, was counted in wells P0 and P1 and summarized.

### Myoblast culture

The first passage was performed on day 11; the cells were passaged from a 6-well plate to a 25 cm^2^ flask, with a seeding density of 1,000 cells/1 cm^2^. The cells were cultured in SKGM-2 or DFEFH medium at 37°C, 5% CO_2_ and 95% humidity. The medium was changed twice a week. The cells were harvested after 7 days and reseeded again at the same density of 1×10^3^ cells/1 cm^2^.

### Manual cell counts to assess myoblast number

Every 7 days, the cells were harvested for reseeding; the cell number was counted using a hematocytometer and a light microscope (Olympus, Tokyo, Japan).

### RNA extraction and reverse transcription

Total RNA was extracted using an RNeasy mini kit (Qiagen, Valencia, CA, USA). The reverse polymerase transcription was performed using reverse transcription reagents (Roche/Applied Biosystems, Foster City, CA, USA) or M-MLV Reverse Transcriptase (Promega, Madison, WI, USA) according to the manufacturer’s instructions.

### Quantitative PCR

Myostatin, Myf5, myogenin and MYH2 expression levels were determined by quantitative (real-time) PCR on an ABI PRISM 7300 Sequence Detection System (Applied Biosystems) using a commercially available TaqMan PCR master mix. Spanning an exon junction, the TaqMan probes used for this analysis were as follows: myostatin (Hs00976237_m1), Myf5 (Hs00271574_m1), myogenin (Hs01032275_m1), and MYH2 (Hs00430042_m1), all from Applied Biosystems. The 2^−ΔΔCT^ method was used to quantify the relative expression levels of the genes. The mRNA expression levels for all samples were normalized to the housekeeping gene, GAPDH.

### Purity evaluation

The percentage of CD56^+^ cells was assessed using monoclonal anti-CD56-APC antibodies (BD Biosciences, San Jose, CA, USA) and cytofluorymetric evaluation on a FACSCalibur with a CellQuest analysis software (BD Biosciences Immunocytometry Systems).

### Evaluation of the fusion potential of MDCs

To evaluate fusion potential, the cells were seeded in 96-well plates in duplicate; once the cells reached 80% confluence, the culture medium was changed to DMEM with 2% FBS and 25 *μ*mol/l insulin, as previously described (40). The medium was changed twice a week. On day 11, the cells were fixed and stained with Wright’s eosin methylene blue solution (Merck, Darmstadt, Germany). The number of nuclei in the 6 largest myotubes was counted in each well.

### Statistical analysis

Statistical analysis was performed using a one- or two-tailed paired Student’s t-test with Microsoft Excel; a value of p<0.05 was considered to indicate a statistically significant difference.

## Results

### Growth of primary myoblast colonies

**Figure 1:**
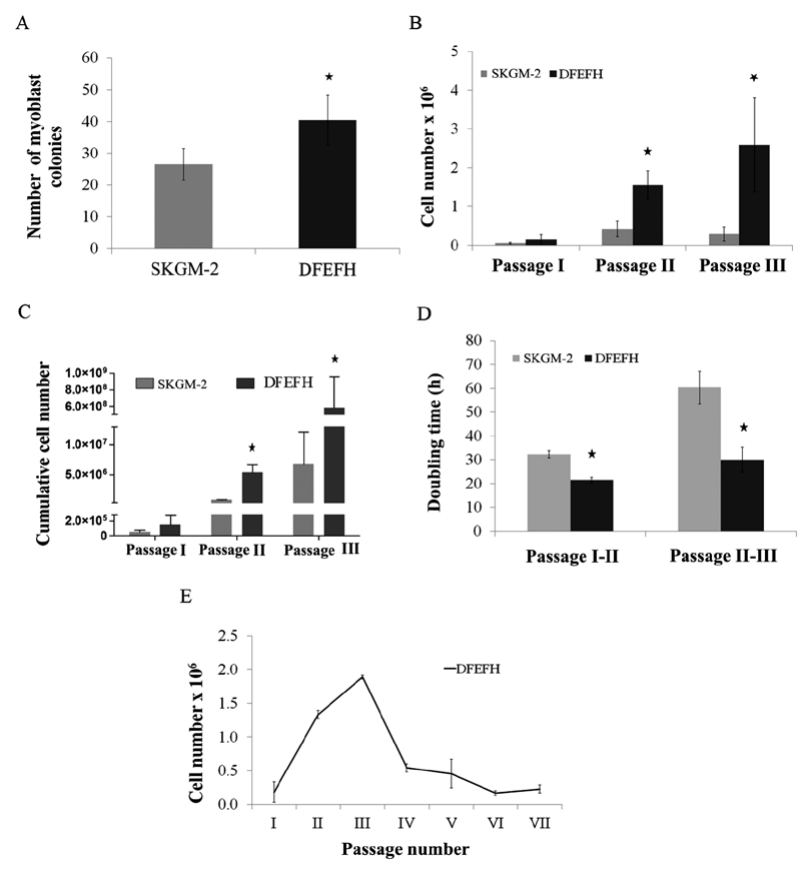
Effect of the culture medium on the number of myoblast colonies, the doubling time and expansion efficacy. Suspensions containing muscle fibers and single cells after collagenase digestion of muscle tissue from approximately 0.02 g of biopsy fragments were seeded on laminin-coated wells of a 6-well plate in skeletal muscle cell growth medium-2 (SKGM-2) or DFEFH medium (P0). After 48 h, supernatants were transferred into new wells (P1), fresh medium was added to P0, and the procedure was repeated after another 48 h. At day 8, the number of colonies, defined as at least 10 cells, was counted in P0 and P1 and added. At day 11, the first passage was performed and the cells were counted. Cells were seeded at a density of 0.025×10^6^/25 cm^2^ flask and passaged with a constant seeding density every 6–7 days until the 3rd passage. The cell number was counted and the doubling time was calculated at each passage. Cells expanded in DFEFH medium were cultured until passage 7, starting with the same seeding density of 0.025×10^6^ cells/25 cm^2^ flask from passage 1 to the final passage. (A) The number of myoblast colonies obtained in different media on day 6, n=2 (2 different donors); ^*^p<0.05. (B) The proliferation rate of myoblast expansion during passages 1–3, n=3 (3 different donors); ^*^p<0.05. (C) The cumulative cells number, that could be obtained in SKGM-2 and DFEFH medium after 3 passages, n=3 (3 different donors); ^*^p<0.05. (D) The doubling time of myoblasts cultured in different medias at passage 2 and 3, n=3 (3 different donors); ^*^p<0.05. (E) The profile of the kinetics of myoblast proliferation in DFEFH medium, n=2 (2 different donors).

To isolate the satellite cells, the digested muscle tissue was seeded in culture medium on laminin-coated plates to enhance the adherence of satellite cells, as previously described (34). At the beginning of myoblast expansion, the same volume of digested muscle tissue was seeded in single wells of a laminin-coated 6-well plate in 2 types of medium, SKGM-2 and DFEFH. On day 6, the number of growing myoblast colonies was counted using an inverted microscope. As shown in Fig. 1A, culture in the DFEFH medium resulted in an average of 41 myoblast colonies compared with 27 myoblast colonies obtained with culture in the SKGM-2 medium. Thus, a statistically significant increase in the colony number was observed.

### Proliferation rate and doubling time of satellite cells

The cells were passaged for the first time after 11 days, when the colonies had begun to be more associated with each other. As shown in Fig. 1B, culture in DFEFH medium resulted in 0.15×10^6^ cells, a number which was approximately 3-fold higher than that of the cells (0.05×10^6^ ) cultured in SKGM-2 medium (Fig. 1B). The cells were seeded into 25 cm^2^ culture flasks with the same density, i.e., 1×10^3^ cells/cm^2^. The following passages were performed once one of the cultures reached approximately 75% confluence, after approximately 6–7 days. In 2 further passages, the cells cultured in DFEFH medium required less time to obtain passaging confluence; consequently, a higher number of cells was obtained with this medium. After the 2nd passage, the difference in the number of cells obtained was even higher, i.e., 1.55×10^6^ cells were obtained with DFEFH medium, a number which was approximately 4-fold higher than that of cells (0.4×10^6^) obtained by culture in SKGM-2 medium. Finally, after the 3rd passage, the greatest difference was observed, with 2.6×10^6^ cells obtained from DFEFH medium cultures, that is, a number which was approximately 9-fold higher than that of cells (0.29×10^6^) cultured in SKGM-2 medium. The differences at passages 2 and 3 were statistically significant (p<0.05). Cumulative cell numbers, which could be finally achieved by seeding all the cells at each passage, were calculated. Cell culture in DFEFH medium obtained 5.84×10^8^ myoblasts, while culture in SKGM-2 obtaiend only 6.7×10^6^ myoblasts (Fig. 1C).

In brief, our results indicated that culture in DFEFH medium, starting with the same volume of digested tissue as with culture in SKGM-2 medium, produced a greater number (100-fold) of myoblasts compared with culture in SKGM-2 medium. The doubling times for the cells cultured in DFEFH medium were 21.5 and 30 h between passages 1 and 2 and between passages 2 and 3, respectively (Fig. 1D). The cells cultured in SKGM-2 medium had doubling times of 32.3 and 60.3 h between passages 1 and 2 and between passages 2 and 3, respectively.

Only the cultures from the DFEFH medium were submitted to further passages; after the 3rd passage, the proliferation rate of the cultures considerably decreased and remained low until the end of the expansion phase at the 7th passage (Fig. 1E).

### Expression of proliferation and differentiation markers by expanded myoblasts

**Figure 2:**
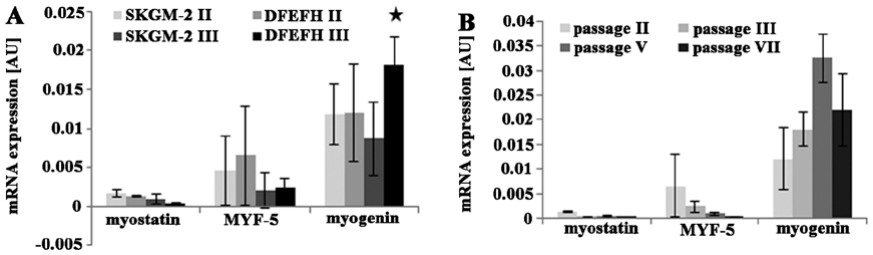
Effect of culture medium on the expression of myogenic genes in cultured myoblasts. Myoblasts were cultured in skeletal muscle cell growth medium-2 (SKGM-2) and DFEFH medium to passage 3. RNA was isolated from the cells at passages 2 and 3, reverse transcribed into cDNA and the expression of myogenic genes was assessed through quantitative analysis. Additionally, cells cultured in DFEFH medium were subjected to higher passages, and the expression of genes was assessed in a similar manner. (A) The expression of mRNA for myostatin, MYF-5, and myogenin genes of human skeletal myoblasts cultured in different medias at passages 2 and 3, n=2 (2 different donors); ^*^p<0.05. (B) The expression of mRNA for myostatin, MYF-5, and myogenin genes of human skeletal myoblasts cultured in DFEFH and subjected to further passages, n=2 (2 different donors).

The mRNA expression levels of the selected genes were investigated by quantitative PCR. The expression levels of the myostatin, Myf5, and myogenin genes were slightly lower at passage 3 than at passage 2, when the myoblasts were cultured in SKGM-2 medium (Fig. 2A). Similar results were observed for myoblasts cultured in DFEFH medium; the expression of the myostatin and Myf5 genes in myoblasts cultured in DFEFH medium was lower at passage 3 than at passage 2. By contrast, the expression of myogenin was higher at passage 3 than at passage 2 for myoblasts cultured in DFEFH medium (Fig. 2A). Furthermore, a comparison of the gene expression levels between myoblasts expanded in the different media, but during the same passage indicated that myostatin expression was 25% lower at passage 2 and approximately 3-fold lower in the cells cultured in DFEFH at passage 3. The expression of Myf5 was similar at both passage 2 and passage 3. For the cells cultured in DFEFH, myogenin expression was approximately 2-fold higher at passage 3 than at passage 2; this result was statistically significant (p<0.05). An analysis of the mRNA expression levels of myoblasts cultured in DFEFH medium from passage 2 up to passage 7 indicated that the myostatin and Myf5 expression levels decreased from passages 2 to 5. The myogenin expression levels increased by approximately 3-fold of those at the initial phase during expansion and then decreased by approximately 30% at the end of the expansion phase (Fig. 2B).

### Morphological analysis of satellite cells in culture and MyHC-2A expression

**Figure 3:**
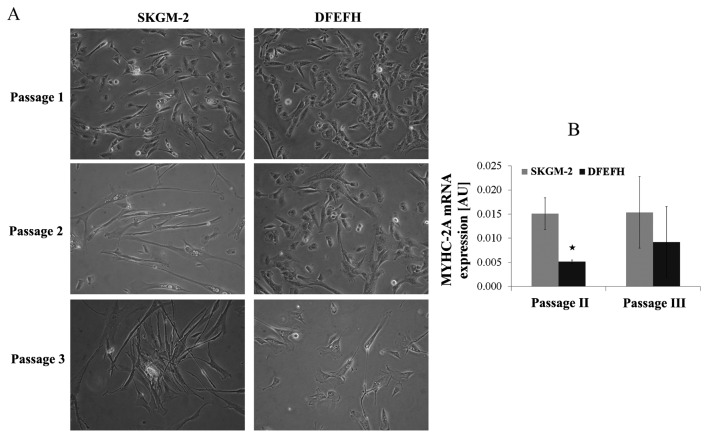
Morphology of myoblasts cultured in different media. Myoblasts were cultured in skeletal muscle cell growth medium-2 (SKGM-2) and DFEFH medium to passage 3. The morphology of the cells was examined before every passage using an inverted microscope. RNA was isolated from the cells at passages 2 and 3, reverse transcribed into cDNA and the expression of the MYHC-2A gene was assessed through real-time analysis. (A) Comparison of the morphology of myoblasts cultured in SKGM-2 and DFEFH medium at passages 1, 2 and 3. Representative results of 2 independent experiments are shown. (B) Comparison of MYHC-2A gene expression in myoblasts cultured in SKGM-2 and DFEFH medium at passages 2 and 3, n=2 (2 different donors).

The morphological aspects of myoblasts cultured in 2 different culture media are shown in Fig. 3A. The morphology of the adherent myoblasts differed between the 2 cultured media. At the beginning of the culture, after passage 1, the difference was subtle. All cells cultured in DFEFH medium were small and spindle-like, whereas some elongated cells in between the spindle-like cells appeared when the cells were cultured in SKGM-2 medium. After the 2nd passage, the difference was considerable. Some elongated cells appeared among the spindle-like cells cultured in DFEFH, but all the cells cultured in SKGM-2 medium became elongated. This considerable difference in the morphology of the cells was retained after the 3rd passage.

All cells expressed MyHC-2A at both the 2nd and 3rd passage. The myoblasts cultured in SKGM-2 medium had a constant, higher expression of MyHC-2A at both passages than those cells cultured in DFEFH (Fig. 3B).

Only the cells cultured in DFEFH medium were submitted to further passage. However, during subsequent passages, most of the cells cultured in DFEFH medium also became elongated and vacuolated (data not shown).

### Purity of myoblast cultures

The average expression of the CD56 antigen in myoblasts cultured in DFEFH medium was 70% (60–80%), while in myoblasts cultured in SKGM-2, it was 55% (20–90%) (data not shown).

### Fusogenic capacity

**Figure 4:**
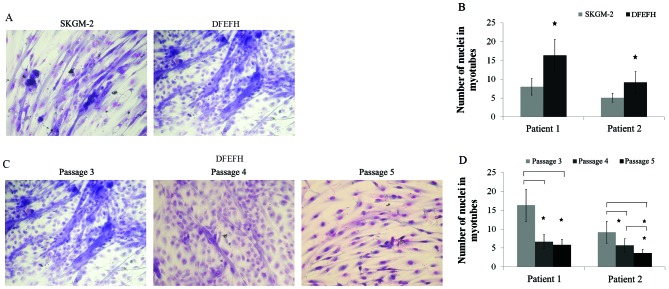
Fusion potential of cultured myoblasts in different media. After passage 3, cells cultured in 2 different media were seeded in duplicate into 96-well plates, and when 80% confluence was obtained, the culture medium was switched to fusion medium [Dulbecco’s modified Eagle’s medium (DMEM), 2% fetal bovine serum (FBS), 25 *μ*mol/l insulin] and changed every 3 days. After 11 days, cells were fixed and stained with Wright’s eosin methylene blue solution. The number of nuclei in the 6 largest myotubes was counted in each well. Additionally, cells cultured in DFEFH medium were subjected to higher passages and their fusogenic potential was assessed in a similar manner. (A) Comparison of the fusogenic potential of myoblasts cultured in skeletal muscle cell growth medium-2 (SKGM-2) and DFEFH medium at passage 3. Representative results of 2 independent experiments are shown. (B) The average number of nuclei in the largest myotubes created from myoblasts expanded in SKGM-2 and DFEFH medium at passage 3, n=2 (2 different donors); ^*^p<0.05. (C) The fusion potential of myoblasts in prolonged cultured in DFEFH medium. Representative results of 2 independent experiments are shown. (D) The average number of nuclei in the largest myotubes created from myoblasts expanded in DFEFH medium at passages 3, 4 and 5, n=2 (2 different donors); ^*^p<0.05.

The formation of myotubes depends entirely on the capacity of the cells to fuse with each other (29). In this study, after the 3rd passage, myoblasts cultured in both types of culture medium had the potential to fuse (Fig. 4A). The average number of nuclei in the 12 randomly selected myotubes was considerably higher for the myoblasts cultured in DFEFH medium (Fig. 4B). For the cells cultured in DFEFH medium, the effect of culture time on fusion potential was also examined. We observed that the fusion potential of the cells markedly decreased with the increasing number of passages, and after passage 5, the fusion potential was very low (Fig. 4C). Moreover, the average number of nuclei in the 12 randomly selected myotubes was considerably lower at passages 4 and 5, in comparison with passage 3 (Fig. 4D).

## Discussion

Myoblasts cultured from small muscle biopsies constitute the basis for evolving cell replacement therapies. Clinical reports on the use of myoblasts for cardiac indications (2,3) and urinary incontinence (1,41) indicate that a high number of cells (≥1×10^8^) is required to obtain therapeutic effects or to improve patient symptoms. Thus, the *in vitro* culture of myoblasts for clinical use aims at obtaining the number of myoblasts with differentiation and fusion potentials that will be sufficient and will be able to regenerate damaged muscle. Culturing myoblasts is challenging as differentiation and myotube formation reduce their ability to grow.

A low-density passaging strategy (1×10^3^cells/cm^2^) has been demonstrated to be the most appropriate for preferable myoblast culture achievement (39). In this study, The commercially available SKGM-2 medium, containing EGF, was compared with DFEFH medium designed by our group, containing EGF, HGF and FGF growth factors.

Our resuls revealed that a) a higher number of satellite cells became activated in the in-house medium, b) their daughter myoblasts had a shorter doubling time and c) their logarithmic growth phase lasted longer than those cells cultured in the commercial medium, SKGM-2. The higher number of myoblast colonies observed in DFEFH medium (Fig. 1A) may indicate that some of the satellite cells present in the muscle biopsy sample may remain inactivated and may be lost when cultured in SKGM-2 medium. DFEFH medium, in contrast to SKGM-2 medium, contained an addition of HGF, shown to be the only factor able to activate these cells *in vivo* and *in vitro* (4,6,7,42). During *in vitro* isolation, mechanical disruption precedes enzymatic digestion. The HGF released during this step may not be sufficient for the activation of all satellite cells; thus, the addition of this activating growth factor may be beneficial for the activation of a higher number of satellite cells.

There was an almost 3-fold higher proliferation rate at the first passage in the DFEFH medium than in the SKGM-2 medium (Fig. 1B), resulting from a greater number (1.6-fold) of colonies, indicating the more rapid growth of the cells in DFEFH medium. The more rapid growth of cells in the DFEFH medium may be a result of stronger proliferation stimulation and/or differentiation prevention. Thus, the proliferation rate of myoblasts obtained by the stimulation of EGF alone in SKGM-2 medium can be enhanced by the stimulation of EGF combined with bFGF and HGF growth factors present in DFEFH medium. We hypothesized that the mechanism responsible for the higher proliferation rate in DFEFH medium may be the result of two additional growth factors, HGF and bFGF. Both HGF and bFGF, present in DFEFH medium, have been shown to increase the *in vitro* proliferation of satellite cells. HGF influences the cells by accelerating cyclin-D1 expression and causing an earlier entry of the cells into the cell cycle (6), while bFGF influences the cells through the stimulation of cyclin-D1 expression (9). Additionally, FGF has been shown to prevent the terminal differentiation of expanded myoblasts through the suppression of MyHC-2A expression (8,11). The addition of insulin to the DFEFH medium, which has been shown to have an anabolic effect on these cells, may be another factor (15).

During the following two passages, 4- and 9-fold higher proliferation rates were observed during the same culture time in DFEFH medium compared with SKGM-2 medium cultures, and each passage began with the same number of cells (Fig. 1B). The peak proliferation rate for myoblasts cultured in SKGM-2 medium was observed at passage 2 after 17 days, while for myoblasts cultured in DFEFH, the peak proliferation rate was observed at passage 3 after 24 days. Different proliferation rates induced different (87-fold higher) cumulative numbers of myoblasts which were obtained from the same biopsy sample in the DFEFH culture. Furthermore, a doubling time of 21.5 and 30 h for the 2nd and 3rd passages, respectively, for the cells cultured in DFEFH medium more closely resembled the 18.2 h observed *in vivo* following the activation of satellite cells in a previous study (42) than the doubling times of 32.3 and 60 h for the 2nd and 3rd passages, respectively, for cells cultured in SKGM-2 medium (Fig. 1D). The doubling time increased after the 2nd passage, at day 17, in both culture media, but for the cells cultured in DFEFH medium, the increase was approximately 50%, whereas for the cells cultured in SKGM-2 medium, the increase was approximately 100%. This result indicates that the composition of DFEFH medium provides the stimulation for cell proliferation throughout the 17 days of culture, resulting in a doubling time similar to the *in vivo* proliferation rate of regenerating muscle cells. To summarize, during the 24 days of culture, myoblasts cultured in SKGM-2 medium underwent approximately 18 doublings, whereas myoblasts cultured in DFEFH medium underwent approximately 25 doublings. Additionally, the average purity of myoblasts cultured in DFEFH medium was greater and more convergent than that of those cultured in SKGM-2 medium. The high diversity of CD56 expression in myoblasts cultured in the previous version of SKGM-2, SKGM, has already been demonstrated (3).

In order to understand how these two culture media influence the endogenous regulation of myoblast proliferation and differentiation, we determined the mRNA expression levels of myostatin during regulation by the myogenic regulatory factors, Myf5 and myogenin, both in myoblasts cultured in DFEFH medium and in myoblasts cultured in SKGM-2 medium.

Molecular analysis revealed that the myostatin expression levels in both populations of cells were 1/4- and 3-fold lower in the cells cultured in DFEFH compared to those cultured in SKGM-2 at passages 2 and 3, respectively (Fig. 2A). This difference may be the result of bFGF, which has been shown to repress the expression of myostatin at the mRNA level, present in the DFEFH medium but absent in the commercial SKGM-2 medium (43). This observation provides a more detailed explanation of the shorter doubling time and the higher proliferation rate of the cells cultured in DFEFH medium as myostatin has been shown to block cell cycle progression by increasing p21 expression, which blocks the release of E2F (25). A decrease in myostatin expression levels between passages 2 and 3 was observed in both media types. However, a greater decrease was observed in myostatin expression levels in DFEFH medium, indicating that the previously described negative autoregulation of myostatin expression (44,45) may occur in order to unblock the differentiation of myoblasts (26,27).

Myf5 expression, confirming the myogenic phenotype of the cells, was detected at passages 2 and 3 in the cells cultured in both media types (46,20). As was expected, during the expansion of the cells, Myf5 expression levels decreased from passage 2 to 3 in both populations. An approximately 50% higher expression of Myf5 was observed at passage 2 in the cells cultured in DFEFH medium compared with those cultured in SKGM-2 medium. However, the higher expression level of Myf5 in cells that underwent two more doublings may be the result of a combination of growth factors that lower myostatin expression in these cells, as myostatin has been shown to inhibit the mRNA expression of Myf5 (26,27). At passage 3, the expression of Myf5 was similar in both populations of cells. Although the difference in myostatin expression was greater at passage 3, the cells cultured in DFEFH medium underwent four more doublings than those cells cultured in SKGM-2 medium; thus, fusion potential, lower myostatin expression levels and lower Myf5 inhibition may balance an expected decrease in Myf5 expression levels during subsequent doublings.

Parallel myogenin expression levels were observed in both populations of cells at the 2nd and 3rd passages, indicating that the cells differentiated into myoblasts (21,47). However, the myogenin expression levels in cells cultured in SKGM-2 medium decreased between passages 2 and 3, indicating that the cells may be entering the early myocyte stage, whereas the myogenin expression levels in cells cultured in DFEFH medium increased between passages 2 and 3 and finally decreased at passage 7 (Fig. 2B), indicating that these conditions may cause the cells to remain at the early differentiating myoblast stage much longer by decreasing myostatin expression levels (47).

Moreover, our data demonstrate that the culture medium composition influences the morphology of the expanded cells (Fig. 3A). Among the small spindle-like myoblasts cultured in SKGM-2 medium, single elongated cells appeared at passage 1, and most of the cells became elongated by passage 2, while the myoblasts cultured in DFEFH medium were still small, spindle-like cells at the second passage. This difference was still apparent at passage 3. However, the myoblasts cultured in DFEFH medium also became elongated and vacuolated at further passages, which correlated with a decreased proliferation rate and an increased doubling time. The differences in morphology may be explained by the difference in the expression levels of the muscle contractile protein, MYHC-2A, which may be associated with phenotypic differentiation (29). MyHC-2A expression was 3-fold and 40% lower at passage 2 and 3, respectively, in myoblasts cultured in DFEFH medium than in those cells cultured in SKGM-2 medium (Fig. 3B). Different morphology could be also partly explained by different purity of myoblasts cultured in different media.

The differentiation potential of the cells in both media was compared at passage 3, when a substantial number of myoblasts could be achieved in both media types, showing similar percentages of cells undergoing fusion. However, the myotubes created from myoblasts expanded in DFEFH medium contained considerably more nuclei after 11 days, which may be explained by the higher purity of the myoblasts culture. The differentiation potential of myoblasts expanded in DFEFH medium was also verified at further passages. A marked decrease in fusion potential was observed after passage 3, leading to an almost complete lack of fusion at passage 5 and a considerable decrease of nuclei in created myotubes. The lack of fusion observed at further passages confirms the recent finding that, with increasing passages, myoblasts become elongated cells with vesicles containing cytoplasm, their differentiation potential decreases and they acquire a different metabolic phenotype (40).

Our results confirm the earlier observations (2,3) that a high number of myoblasts can be obtain in an *in vitro* setting. However, our results also indicate a considerably higher, up to 5×10^9^, expansion efficacy, in a considerably shorter time, i.e., 24 days [in contrast to 1×10^9^ in 46 days after a 0.6–1.9 g biopsy (2), 3×10^8^ in 18 days after a 2 g biopsy (3), or 3×10^8^ in 25 days after a 0.2 g biopsy], which may be caused by biopsy weight, growth factor composition and seeding density (39). We also confirm the findings of earlier reports showing that further expansion results in obtaining myoblasts that contain vacuoles and are unable to fuse (40).

The present study also suggests that conditions enabling the growth of up to 5×10^9^ of myoblasts in 24 days present fusion potential in elderly patients. Additionally, it shows that the expansion of the cells over 23 generations results in the rapid decrease of proliferation potential, an increase in doubling time, the appearance of vacuoles in cells and the loss of fusion potential.

Setting standard conditions for the medium composition of myoblast expansion for clinical purposes by manipulating the intrinsic control of proliferation and differentiation may be of value. For the clinical transplantation of functionally competent cells for successful cell therapy and the unnecessary costs of prolonged cultures, the maximal number of doublings, rather than passages, that cells can undergo in *in vitro* conditions should be set.

## Abbreviations

SKGM-2: skeletal muscle cell growth medium-2
MDCs: muscle-derived cells
HGF: hepatocyte growth factor
bFGF: basic fibroblast growth factor
EGF: epidermal growth factor
PDGF: platelet-derived growth factor
TGFβ: tumor growth factor-β
DMEM: Dulbecco’s modified Eagle’s medium
PBS: phosphate-buffered saline
FBS: fetal bovine serum
